# The Delta Subunit of Rod-Specific Photoreceptor cGMP Phosphodiesterase (PDE6D) Contributes to Hepatocellular Carcinoma Progression

**DOI:** 10.3390/cancers11030398

**Published:** 2019-03-21

**Authors:** Peter Dietrich, Claus Hellerbrand, Anja Bosserhoff

**Affiliations:** 1 Institute of Biochemistry, Emil-Fischer-Zentrum, Friedrich-Alexander-University, Erlangen-Nürnberg, 91054 Erlangen, Germany; peter.dietrich@fau.de (P.D.); anja.bosserhoff@fau.de (A.B.); 2 Department of Medicine 1, University Hospital Erlangen, Friedrich-Alexander-University, Erlangen-Nürnberg, 91054 Erlangen, Germany; 3 Comprehensive Cancer Center (CCC) Erlangen-EMN, 91054 Erlangen, Germany

**Keywords:** HCC, KRAS, PDE6D, sorafenib, TGF-β

## Abstract

Emerging evidence reveals crucial roles of wild type RAS in liver cancer. The delta subunit of *rod-specific photoreceptor cGMP phosphodiesterase* (PDE6D) regulates the trafficking of RAS proteins to the plasma membrane and thereby contributes to RAS activation. However, the expression and specific function of PDE6D in hepatocellular carcinoma (HCC) were completely unknown. In this study, PDE6D was newly found to be markedly upregulated in HCC tissues and cell lines. Overexpression of PDE6D in HCC correlated with enhanced tumor stages, tumor grading, and ERK activation. PDE6D depletion significantly reduced proliferation, clonogenicity, and migration of HCC cells. Moreover, PDE6D was induced by TGF-β1, the mediator of stemness, epithelial-mesenchymal transition (EMT), and chemoresistance. In non-resistant cells, overexpression of PDE6D conferred resistance to sorafenib-induced toxicity. Further, PDE6D was overexpressed in sorafenib resistance, and inhibition of PDE6D reduced proliferation and migration in sorafenib-resistant HCC cells. Together, PDE6D was found to be overexpressed in liver cancer and correlated with tumor stages, grading, and ERK activation. Moreover, PDE6D contributed to migration, proliferation, and sorafenib resistance in HCC cells, therefore representing a potential novel therapeutic target.

## 1. Introduction

Hepatocellular carcinoma (HCC) is one of the most rapidly rising causes of cancer-related mortality [1,2]. Current treatment options using sorafenib as a first line [3,4,5] and regorafenib as a second line [6] strategy are the only approved systemic therapeutic options for advanced HCC. These synthetic small molecule inhibitors target RAS/RAF/ERK signaling in HCC cells, thereby underlining the prominent function of RAS downstream pathways in liver cancer [3,7,8].

Recently, our group showed that wild type RAS proteins (including Kirsten rat sarcoma (KRAS) and HRas proto-oncogene (HRAS)) are promising novel microRNA-regulated therapeutic targets and contribute to acquired resistance to RAF inhibitors in HCC [9,10] and melanoma [11,12].

The delta subunit of *rod-specific photoreceptor cGMP phosphodiesterase* (PDE6D) is a ubiquitously expressed prenyl-binding protein [13,14,15]. By binding to prenyl moieties, PDE6D mediates the shuttling of prenylated proteins between membranes [16]. Thereby, it regulates the membrane association of Ras and Rap GTPases [16].

Recently, PDE6D was found to mediate the antegrade trafficking of prenylated KRAS to the cell membrane, where KRAS is activated [17,18,19,20]. Novel small molecule inhibitors (i.e., “deltarasin” and “deltazinone 1”), which bind to the prenyl-binding pocket of PDE6D, impair the enrichment of RAS proteins at the plasma membrane [17,21], thereby inhibiting RAS activation, which has been considered a desirable goal in cancer research for a long time. Meanwhile, studies by our and other groups suggested that targeting PDE6D-KRAS interaction is an effective approach to tackle both KRAS wild type [9,11] and -mutated [17,21,22,23,24] cancer types. Apart from these studies pointing to a major role of PDE6D-KRAS interaction in cancer, PDE6D has been found to be significantly expressed in human breast cancer cells and tissues and was considered to play a role in transducing the effects of light on breast cancer [25]. Moreover, PDE6D was found to be a potential novel biomarker for colorectal cancer [26]. However, despite these infrequent studies, the expression, precise function, and cellular localization of PDE6D in cancer including HCC, as well as its potential role in therapy resistance, remained unknown and were addressed in this study.

## 2. Results

### 2.1. Expression of PDE6D in Hepatocellular Carcinoma

**Figure 1:**
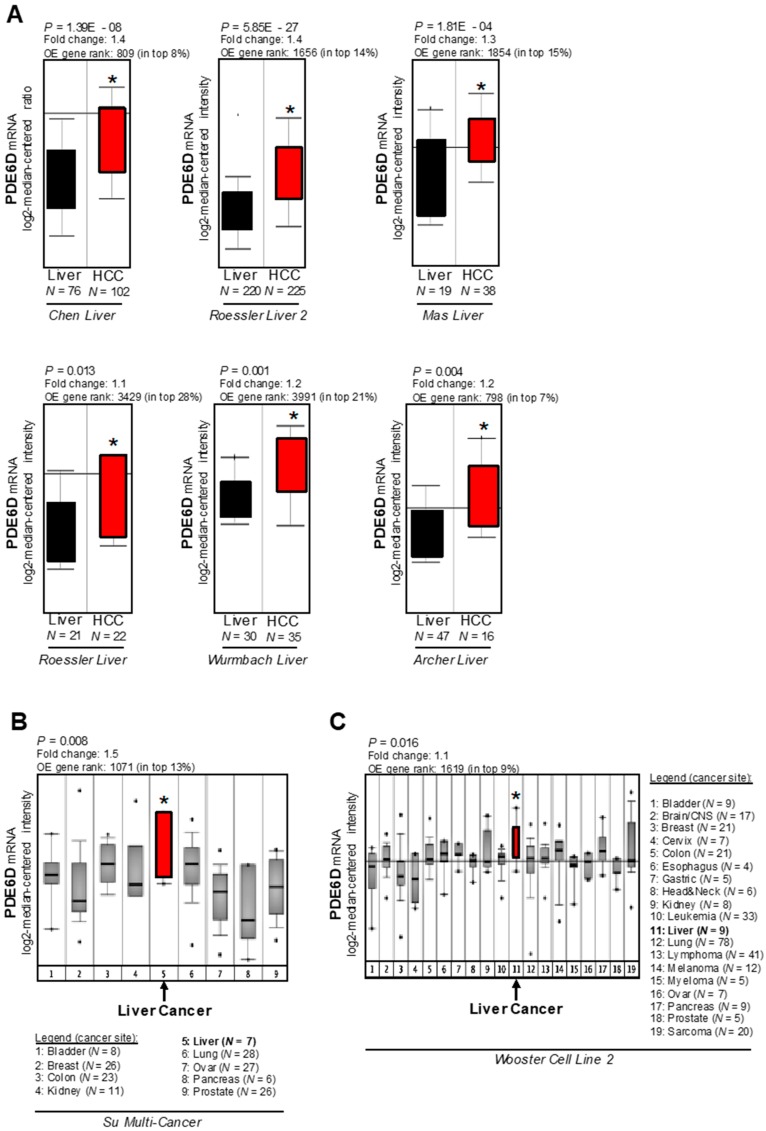
In silico analysis of *rod-specific photoreceptor cGMP phosphodiesterase* (PDE6D) expression in hepatocellular carcinoma (HCC). (**A**) Oncomine^TM^ human cancer microarray database analysis of six patient datasets depicting PDE6D mRNA expression levels in HCCs and non-tumorous livers (* *p* < 0.05 vs. non-tumorous livers). (**B**,**C**) Oncomine^TM^ human cancer microarray database analysis of PDE6D expression as detected in large-scale RNA profiling studies comparing diverse carcinomas of different origins (* *p* < 0.05 vs. average expression).

**Figure 2:**
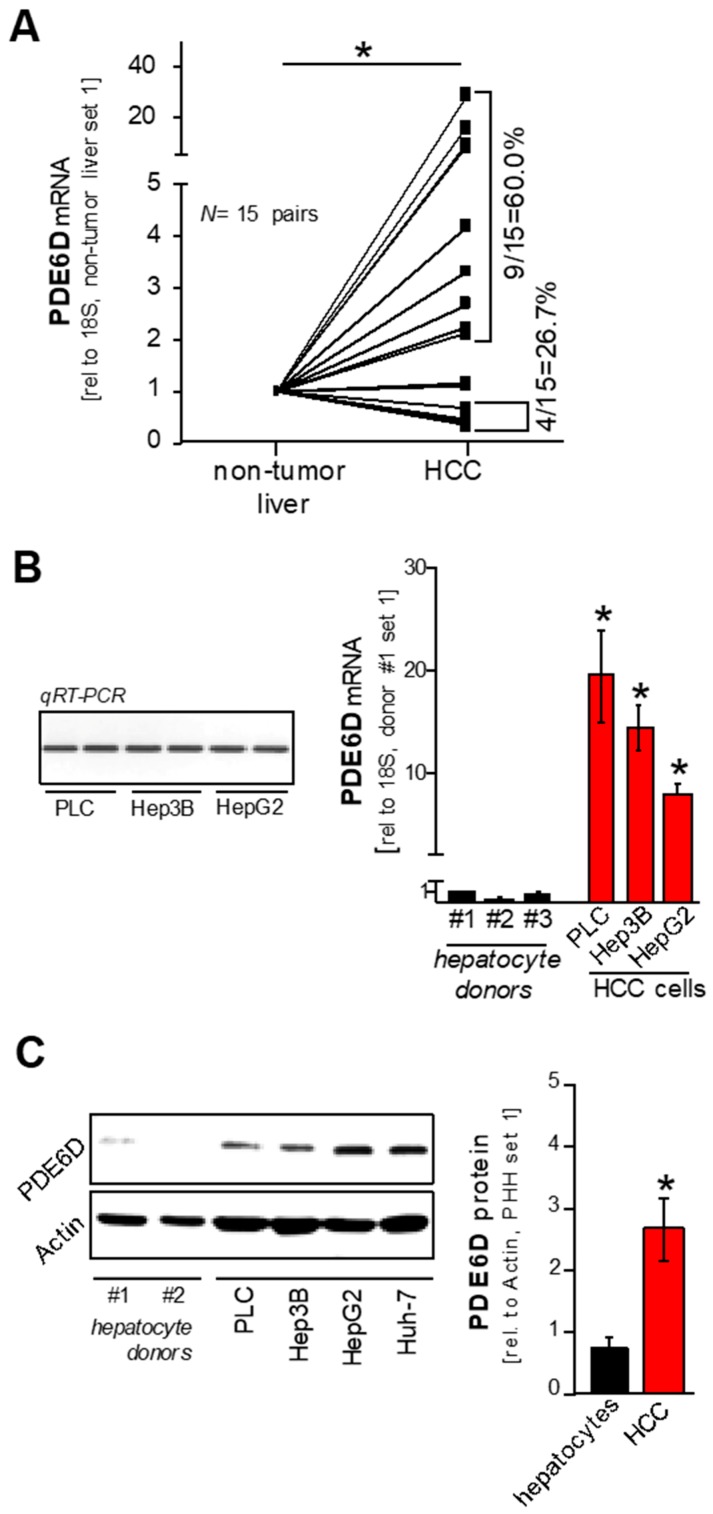
PDE6D expression in HCC in vivo and in vitro. (**A**) PDE6D mRNA levels as quantified by qRT-PCR analysis of HCC patient samples and paired non-tumorous liver tissues (* *p* < 0.05). (**B**) Detection of PDE6D mRNA in human HCC cells (PLC, Hep3B, HepG2) after qRT-PCR amplification using gel electrophoresis (left panel) and relative PDE6D mRNA levels (qRT-PCR) in human HCC cell lines (PLC, Hep3B, HepG2) compared with primary human hepatocytes derived from different donors (#1–3) (right panel) (* *p* < 0.05 vs. hepatocytes). (**C**) Exemplary Western blot image (left panel) and summarized densitometric quantification (right panel) of PDE6D protein levels in HCC cells (PLC, Hep3B, HepG2, Huh-7) compared with hepatocytes derived from different donors (#1–2) (* *p* < 0.05 vs. hepatocytes).

PDE6D promotes RAS signaling by mediation of the antegrade trafficking of KRAS to the cell membrane. We showed previously that wild type RAS proteins are upregulated in HCC [9,10] and therefore hypothesized that PDE6D (over)expression might contribute to HCC progression. In silico analysis was performed using the Oncomine^TM^ human cancer microarray database [27] to determine PDE6D expression in HCC patient tissues compared to non-tumorous livers. PDE6D expression levels were strongly upregulated in several HCC patient datasets (“Roessler Liver” [28]; “Roessler Liver 2” [28]; “Wurmbach Liver” [29]; “Chen Liver” [30]; “Mas Liver” [31]; “Archer Liver” [32]) (Figure 1A). Moreover, datasets deriving from large-scale RNA profiling studies aimed at characterizing molecular classification schemes for diverse carcinomas [33] revealed significant overexpression of PDE6D in liver cancer as compared to multiple other cancer types (Figure 1B,C). Next, we aimed to confirm this marked overexpression of PDE6D in human HCC in vitro and in vivo. Quantitative RT-PCR analysis of paired tumor specimens and corresponding non-tumorous liver tissues revealed significant upregulation of PDE6D mRNA levels in HCC tissues (*N* = 15 pairs) (Figure 2A). Furthermore, PDE6D mRNA was detected in human HCC cell lines (Figure 2B), and both PDE6D mRNA and protein levels were strongly overexpressed in HCC cells as compared to primary human hepatocytes derived from different donors (Figure 2B,C). In summary, these data revealed that PDE6D is markedly overexpressed in human HCC, pointing to potential oncogenic functions of this KRAS-transport chaperon.

### 2.2. PDE6D Effects on HCC Proliferation, Clonogenicity and Sorafenib Resistance

**Figure 3:**
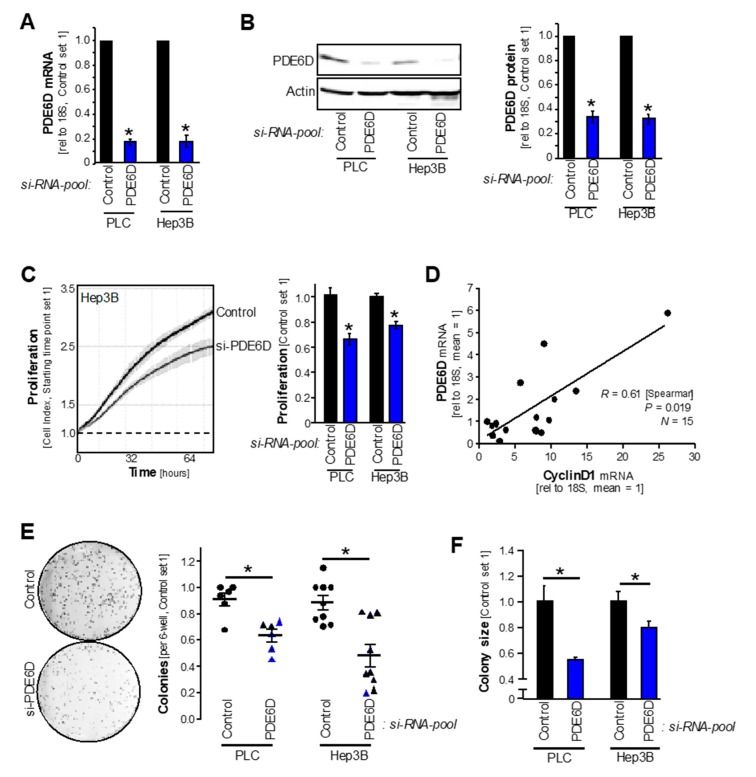
Effects of PDE6D knockdown on HCC proliferation and clonogenicity. Prior to functional experiments, HCC cell lines (PLC, Hep3B) were transfected with *si-RNA-pools* against PDE6D (“PDE6D”) or the according control-*si-RNA-pool* (“Control”). (**A**) PDE6D mRNA levels as quantified by qRT-PCR analysis (* *p* < 0.05 vs. control). (**B**) PDE6D protein levels as quantified by Western blot analysis. The left panel depicts an exemplary Western blot image, and the right panel depicts the summarized densitometric quantification (* *p* < 0.05 vs. control). (**C**) Real-time cell proliferation (xCELLigence). Exemplary proliferation curves (left panel) and quantified “slopes” (summarizing the proliferative ability) (right panel) are shown (* *p* < 0.05 vs. control). (**D**) Relative (to mean) PDE6D as correlated to CyclinD1 mRNA expression levels (qRT-PCR) in human HCC patient tissue samples. (**E**,**F**) Anchorage-dependent clonogenic assay (an exemplary image (Hep3B) is depicted in the left panel of (**E**)). Quantification of colony number (right panel of (**E**)) and sizes (**F**) (* *p* < 0.05).

To decipher the functional role of PDE6D in HCC, *si-RNA-pool*-mediated knockdown experiments were performed (“si-PDE6D”: functionally verified pool of 30 single si-RNAs against human PDE6D; an according *si-RNA-control-pool* was used to control for off-target effects). Knockdown of PDE6D mRNA and protein levels was established in HCC cell lines (Hep3B, PLC) (Figure 3A,B). Real-time cell proliferation analysis revealed strong reduction of proliferation after *si-RNA-pool*-mediated PDE6D inhibition as compared to control-treated cells (Figure 3C). Accordingly, qRT-PCR analysis showed a significant correlation of PDE6D expression with the proliferation marker CyclinD1 in human HCC patient tissues (*R* = 0.61, *P* = 0.019, *N* = 15) (Figure 3D). Next to proliferation, clonogenicity assays revealed that PDE6D knockdown strongly reduced the number and size of colonies formed by HCC cells (Figure 3E,F). In accordance with the finding that inhibition of PDE6D impairs the enrichment of RAS proteins at the plasma membrane [17,21], we found that PDE6D suppression (using si-RNA technique and pharmacologic inhibition of the PDE6D-KRAS interaction) reduced MAPK-ERK activation in HCC cells (Figure S1A,B).

**Figure 4:**
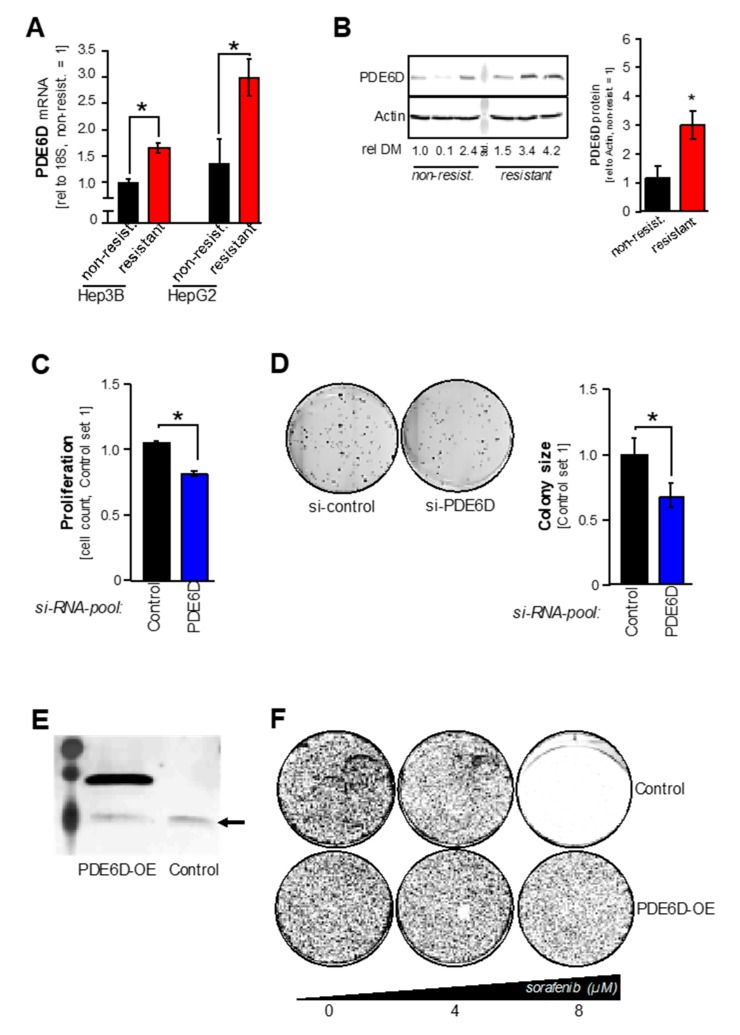
Expression and function of PDE6D in sorafenib resistance. (**A**) PDE6D mRNA levels as quantified by qRT-PCR analysis in non-resistant (“non-resist.”) as compared to sorafenib-resistant (“resistant”) Hep3B and HepG2 cell clones (* *p* < 0.05). (**B**) Exemplary image (left panel) and summarized densitometric quantification (right panel) of Western blot analysis of PDE6D levels in non-resistant (“non-resist.”) as compared to sorafenib-resistant (“resistant”) Hep3B cells (rel DM: relative (PDE6D/Actin) densitometry) (* *p* < 0.05 vs. non-resist.). (**C**,**D**) Prior to functional experiments, resistant cells (Hep3B) were transfected with *si-RNA-pools* against PDE6D (“PDE6D”) or the according control-*si-RNA-pool* (“Control”). (**C**) Depicts relative proliferation (cell numbers) and (**D**) depicts exemplary clonogenic assays. (**E**,**F**) Forced overexpression of PDE6D protein (PDE6D-OE) in HCC cells (e.g., PLC) was performed by transfection of a human PDE6D open reading frame (ORF) Myc-DDK-tagged plasmid vector (an empty control vector without the PDE6D ORF was used as control treatment). (**E**) Depicts Western blot analysis depicting the overexpressed Myc-DDK-tagged PDE6D protein after PDE6D-OE as well as endogenous (arrow) PDE6D in both PDE6D-OE and control-treated cells. (**F**) Depicts exemplary images (representing 8 replicate values of 2 independent experiments) of cells cultured in 6-wells for 72 h (100,000 cells were initially seeded per 6-well) and treated with different doses of sorafenib (0, 4, 8 µM).

Recent studies by our group have identified a crucial role of wild type KRAS in sorafenib-resistance [9]. Therefore, we asked if PDE6D might also contribute to sorafenib resistance in HCC cells. Long-term exposure to slowly increasing doses of sorafenib was performed to establish sorafenib-resistant Hep3B and HepG2 cell clones. Quantitative RT-PCR and Western blot analysis revealed significant upregulation of PDE6D mRNA and protein levels in sorafenib-resistant as compared to non-resistant HCC cell clones (Figure 4A,B). Functionally, *si-RNA-pool*-mediated PDE6D knockdown reduced proliferation (Figure 4C) and clonogenicity (Figure 4D) in resistant HCC cell clones. These effects suggested that PDE6D might functionally induce sorafenib resistance. Accordingly, additional overexpression of PDE6D protein (Figure 4E) in non-resistant cells rescued sorafenib-induced toxicity (Figure 4F). In contrast to PDE6D knockdown and/or inhibition, forced PDE6D overexpression induced ERK activation in resistant HCC cells (Figure S1C), which might contribute to enhanced resistance to the unspecific RAF inhibitor sorafenib. Likewise, combined treatment using PDE6D inhibition and sorafenib was proven to enhance apoptosis in resistant HCC cells [9]. Together, PDE6D mediated proliferation, clonogenicity, and ERK activation in HCC. Moreover, our data point to a functional role of the PDE6D-KRAS axis in acquired sorafenib resistance in HCC.

### 2.3. TGF-β-Mediated Regulation of PDE6D in HCC

**Figure 5:**
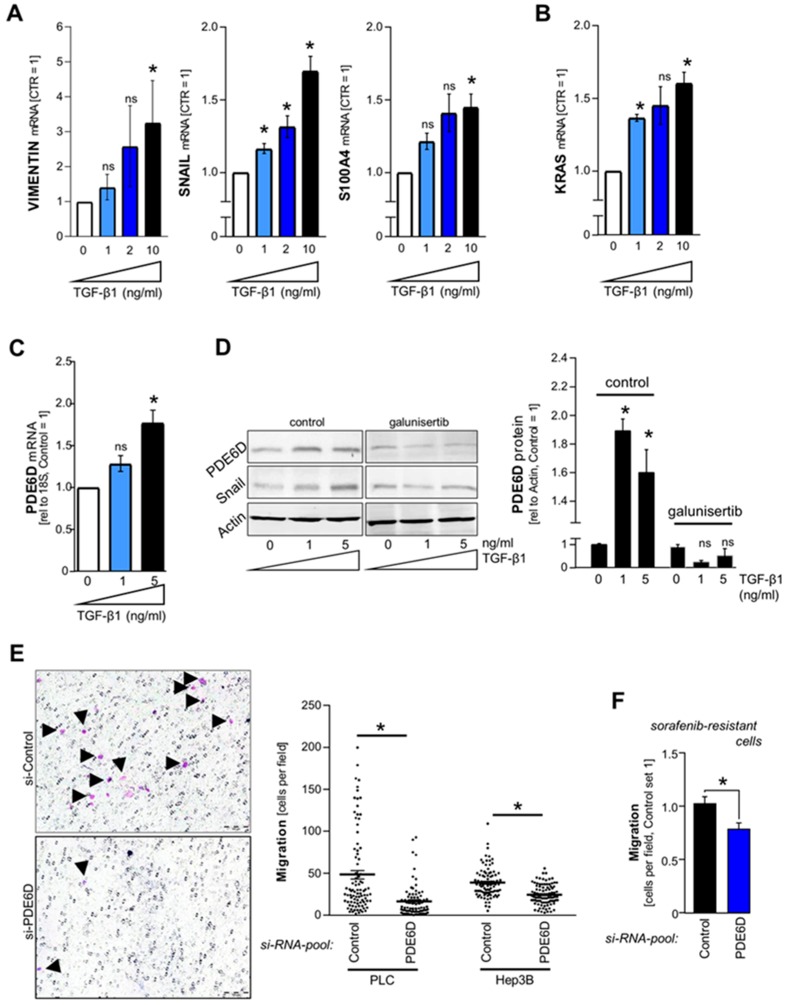
TGF-â-mediated regulation of PDE6D and the effect of PDE6D on HCC cell migration. (**A**–**C**) Quantitative RT-PCR analysis of VIMENTIN, SNAIL, S100A4 (**A**) and KRAS (**B**) mRNA levels in HCC cells (PLC) that were stimulated with different doses of recombinant human TGF-â1 protein for 72–96 hours (* *p* < 0.05 vs. control). (**C**,**D**) Quantitative RT-PCR revealing mRNA levels (**C**) as well as protein levels as quantified by Western blot analysis (including a representative Western blot image) (the densitometric values represent two independent Western blot analysis) (**D**) of PDE6D expression in HCC cells (PLC) that were treated with different doses of recombinant human TGF-â1 for 72–96 hours. (**D**) also depicts co-treatment with 15 µM of the TGF-â-receptor-1 (TGFBR1) inhibitor LY2157299 (“galunisertib”) (* *p* < 0.05 vs. control). (**E**,**F**) Prior to Boyden chamber experiments, non-resistant HCC cells (PLC, Hep3B) (**E**) and sorafenib-resistant Hep3B cells (**F**) were transfected with *si-RNA-pools* against PDE6D (“PDE6D”) or the according control-*si-RNA-pool* (“Control”). Migration (migrating cells per visual field) as measured by Boyden chamber migration assay (duration of migration: 4 hours) is depicted as absolute cell counts (**E**) or as normalized migration (**F**) (* *p* < 0.05).

Transforming growth factor-β (TGF-β)-signaling and TGF-β-mediated epithelial-mesenchymal transition (EMT) were recently shown to induce sorafenib resistance by induction of receptor tyrosine kinases and stemness features in HCC cells [34]. The effects of PDE6D on stemness-related clonogenicity (Figure 3) and sorafenib resistance (Figure 4) prompted us to explore whether TGF-β might be involved in the regulation of PDE6D in HCC. TGF-β1 treatment dose-dependently induced expression of HCC-related EMT markers [35], including Vimentin, Snail, and S100A4 in HCC cells (Figure 5A). In our previous study, we found that wild type KRAS (partly) regulated the expression of several EMT markers in HCC [9]. Here, we showed that conversely, TGF-β1-treatment also induced KRAS expression in HCC cells (Figure 5B). Furthermore, upregulation of PDE6D mRNA and protein levels was also mediated by TGF-β1 (Figure 5C,D). The TGF-β-receptor-1 (TGFBR1) inhibitor LY2157299 (“galunisertib”) (currently investigated as potential therapeutic option in HCC in clinical trials [36,37]) antagonized TGF-β1-mediated upregulation of Snail and PDE6D (Figure 5D; Figure S2). In summary, TGF-β-mediated induction of EMT markers was accompanied by co-upregulation of KRAS and PDE6D in HCC, which might be involved in sorafenib resistance and stemness features including clonogenicity.

### 2.4. Effect of PDE6D on HCC Cell Migration

Next to the known effects of TGF-β signaling on EMT, stemness, clonogenicity and sorafenib resistance, TGF-β1-mediated enhanced migration has been described in HCC [38,39]. However, in our previous study, the migratory potential of HCC cells was not affected by KRAS suppression [9]. Here, Boyden chamber assays unexpectedly revealed that PDE6D inhibition strongly impaired migration in HCC cells (PLC, Hep3B) (Figure 5E). Similarly, the migration of sorafenib-resistant HCC cells was reduced after PDE6D knockdown (Figure 5F). Taken together, PDE6D inhibition impaired migration in both non-resistant and sorafenib-resistant HCC cells, while it was recently shown that KRAS did not affect migration. These data pointed to additional functions of PDE6D in HCC beyond its role in mediation of KRAS trafficking to the plasma membrane.

### 2.5. Cellular Localization and Expression of PDE6D in HCC

**Figure 6:**
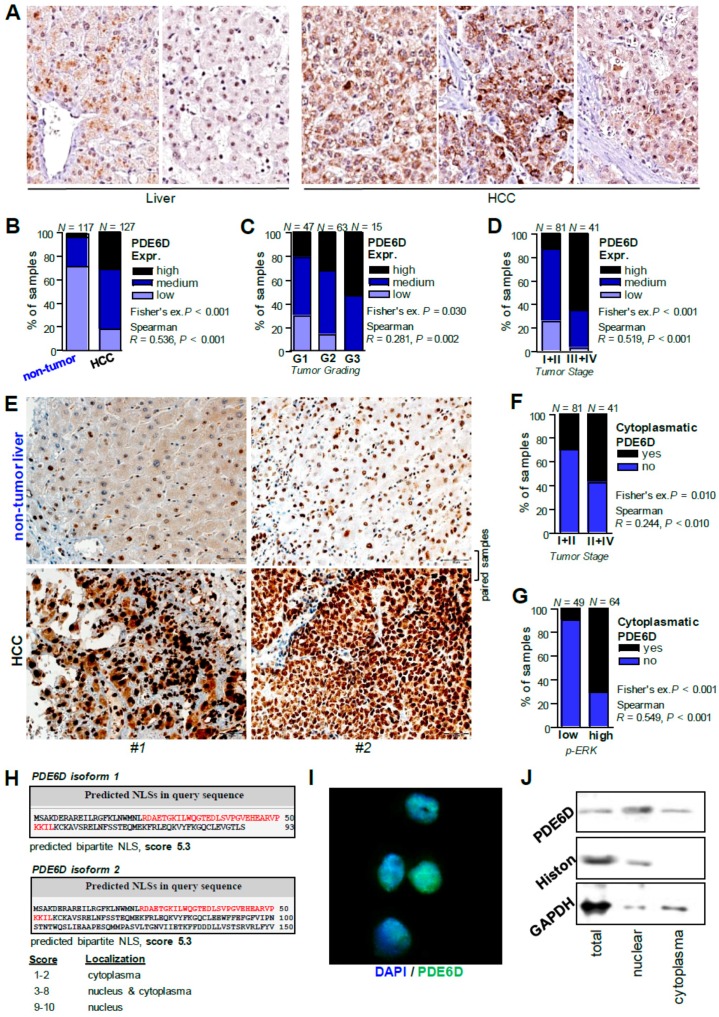
Expression and cellular localization of PDE6D in HCC in vivo and in vitro. (**A**) PDE6D staining (exemplary images) of non-tumorous liver tissues (left side) and HCC tissues (right side) deposited on the Human Protein Atlas database. (**B**) Tissue microarray analysis of PDE6D expression levels in human HCC tissues (*N* = 117) as compared with corresponding non-tumorous liver tissues (*N* = 127) (Fisher’s exact *P* < 0.001). (**C**,**D**) Tissue microarray analysis of PDE6D expression levels in human HCC tissues correlated with tumor grading (Fisher’s exact *P* = 0.030) (**C**) and tumor stages (**D**). (**E**) Exemplary immunohistological images of PDE6D protein expression in human HCC samples and corresponding non-tumorous liver tissues applying a tissue microarray revealing nuclear staining next to cytoplasmatic staining patterns (paired samples of two different patients (#1, #2) are depicted). (**F**,**G**) Tissue microarray analysis comparing tumor stages (**F**) and ERK activation (p-ERK) (**G**) in human HCC tissues with (“yes”) and without (“no”) cytoplasmatic localization pattern of PDE6D. (**H**) In silico-based analysis of importin-á-dependent nuclear localization signals (NLS, *red letters*) using the “cNLS Mapper” predicted bipartite NLS in both isoforms of PDE6D (score for both isoforms was 5.3). A legend depicts that higher scores indicate stronger NLS activity and defines major localizations in dependence of each score. (**I**,**J**) Exemplary immunofluorescence (**I**) and Western blot analysis (**J**) depicting nuclear localization of PDE6D (I) and expression of PDE6D in both nuclear and cytoplasmatic fractions (**J**) of HCC cell lysates.

The effects of PDE6D on migration prompted us to explore the cellular localization of PDE6D in HCC. Moreover, we also aimed to confirm marked overexpression of PDE6D protein expression in human HCC tissues using immunohistochemistry. First, immunostaining of PDE6D in human tissues was explored using the Human Protein Atlas database (https://www.proteinatlas.org/ENSG00000156973PDE6D/pathology#top). In this dataset, stronger expression of PDE6D was found in HCC compared to non-tumorous livers (Figure 6A). Immunohistochemistry analysis of tissue microarrays comprising human HCC tissues [9,40,41] confirmed marked PDE6D overexpression in HCC (Figure 6B). Moreover, higher PDE6D expression correlated with enhanced tumor grading (Figure 6C), tumor stages (Figure 6D), and KRAS expression levels (Figure S3A).

Interestingly, next to the expected cytoplasmatic localization, positive nuclear staining of PDE6D was detected in HCC (Figure 6E). Aiming at characterizing the potential prognostic and functional relevance of different cellular localization patterns of PDE6D, we found that cytoplasmatic PDE6D expression (compared with samples with “nuclear staining only”) was associated with enhanced tumor stages (Figure 6F). Functionally, and in strong accordance with the finding that cytoplasmatic PDE6D can mediate antegrade trafficking of prenylated KRAS to the cell membrane, we found that cytoplasmatic PDE6D expression correlated with positive KRAS membrane staining (representing “activated” KRAS) (Figure S3B) and ERK activation (Figure 6G).

Next to KRAS-MAPK-ERK signaling, the positive nuclear staining pattern pointed to potential additional functions of PDE6D in HCC. In silico-based analysis of importin-α-dependent nuclear localization signals using the “cNLS Mapper” [42,43] predicted bipartite NLS in both isoforms of PDE6D (Figure 6H). The cut-off score for both isoforms was 5.3, defining the NLS to induce partial nuclear and cytoplasmatic localization (Figure 6H), strongly resembling the Human Protein Atlas derived data. Accordingly, nuclear localization of PDE6D in HCC cells (Hep3B, PLC) was detected in vitro using immunofluorescence technique (Figure 6I). Nuclear and cytoplasmatic subcellular fractioning of HCC cell lysates and subsequent Western blot analysis confirmed both nuclear and cytoplasmatic expression of PDE6D (Figure 6J). In silico analysis of protein-protein interaction datasets using the “Harmonizome” database [44] revealed a list of 34 PDE6D-interacting proteins including KRAS, HRAS, and several transcription factors that are known to be involved in HCC progression such as E2F1 [45,46] and HNF4A [47,48] (Figure S4A). Gene enrichment-based on Kyoto Encyclopedia of Genes and Genomes (KEGG) pathway analysis of the 34 interacting proteins for PDE6D revealed significant pathway enrichments associated with cancer including “MAPK signaling” (*P* = 0.00023), “Ras signaling” (*P* = 0.0011), and “TGFβ signaling” (*P* = 0.00623) (Figure S4B). Further gene enrichment analysis using the “Enrichr” database [49] revealed several significantly enriched transcription factor terms for the 34 PDE6D-interacting protein list in two datasets (“Enrichr Submissions TF-Gene Coocurrence” and “Transcription Factor PPIs”) pointing particularly to involvement of p53 and Smad signaling pathways (Figure S5A,B). These findings suggested diverse cytoplasmatic and nuclear functions and potential transcription factor interactions of PDE6D. Together with its effects on migration that were not shown for KRAS in HCC, these data pointed to additional roles of PDE6D beyond its effect on KRAS.

## 3. Discussion

The delta subunit *of rod-specific photoreceptor cGMP phosphodiesterase* (PDE6D) mediates antegrade trafficking of KRAS to the cell membrane [17,18,19,20] implicating its potential crucial role in cancer. Moreover, PDE6D was described to be strongly expressed in breast cancer [25] and found to be a novel candidate relapse biomarker in colorectal carcinoma [26]. However, the function of PDE6D in most cancer types remained elusive, and despite our study suggesting that interruption of the KRAS-PDE6D interaction in liver cancer might represent a novel therapeutic strategy [9], the expression and function of PDE6D in HCC was completely unknown.

Regarding KRAS overexpression in HCC [9], we newly showed that PDE6D mRNA and protein levels are strongly upregulated in HCC cells and tissues. The strong overexpression in HCC points out that PDE6D might further serve as a potential candidate biomarker in HCC as suggested for colorectal carcinoma [26]. Accordingly, we found that PDE6D expression correlated with tumor grading and tumor stages. Moreover, PDE6D expression correlated significantly with KRAS expression levels in HCC tissues, which is in accordance with the finding that PDE6D serves as a novel targetable KRAS transport chaperon in cancer cells.

Inhibition of PDE6D revealed marked reduction of proliferation, clonogenicity and ERK-activation in HCC. PDE6D localization patterns in human HCC tissues revealed that a cytoplasmatic PDE6D expression pattern was associated with enhanced membrane localization of KRAS (which resembles KRAS-activation), enhanced ERK-activation and advanced tumor stages.

However, the effects of *si-RNA*-mediated PDE6D inhibition on proliferation and clonogenicity were not as strong as the effects of direct KRAS inhibition or small molecule-mediated inhibition of the PDE6D-KRAS interaction as performed in our previous study [9]. This could be due to the high expression of PDE6D and thus incomplete knockdown efficacy by si-RNA technique (Figure 3A,B): levels of ~30–40% remaining mRNA and protein expression after PDE6D knockdown in HCC cells were still higher than the expression of PDE6D in hepatocytes. Moreover, regarding KRAS trafficking, other (yet unknown) potential membrane-trafficking chaperons could rescue the inhibition of PDE6D. Still, (incomplete) PDE6D knockdown was sufficient to significantly block proliferation and stemness features in HCC cells. Together with novel and specific highly-efficient small molecule inhibitors of the PDE6D-KRAS interaction [9,17,21], this marks a promising therapeutic strategy for HCC and other cancer types [11]. Furthermore, we identified higher expression of PDE6D in resistant as compared to non-resistant cell lines. PDE6D knockdown reduced proliferation in resistant HCC cell clones, and overexpression of PDE6D in non-resistant cells induced resistance to sorafenib-mediated toxicity, suggesting that PDE6D might also represent a therapeutic target in this patient cohort.

TGF-β signaling and TGF-β-mediated EMT was shown to induce sorafenib resistance by induction of receptor tyrosine kinases and stemness features in HCC [34]. Moreover, TGF-β1-mediated enhanced migration has been described in HCC before [38,39]. Here, we newly found that TGF-β1 signaling upregulated PDE6D expression, and PDE6D inhibition reduced migration in both non-resistant and sorafenib-resistant HCC cells. This finding underscores the potential beneficial effect of galunisertib in the treatment of HCC, as investigated in current clinical trials. Therefore, the question of which intracellular downstream effector(s) of TGF-beta receptor activation (e.g., Smad) could mediate PDE6D upregulation should be addressed in future studies.

Interestingly, we have shown recently that in contrast to PDE6D, KRAS does not affect migration in HCC [9]. Here, we revealed (in addition to its known cytoplasmatic localization) nuclear localization of PDE6D protein in HCC, and in silico analysis pointed to several potential protein-protein interactions of PDE6D with known HCC-related transcription factors including E2F1, HNF4A, and MYC. Moreover, this PDE6D interactome was significantly involved in major cancer-related transcription factor and cytoplasmatic signaling pathways including TGF-β and Smad signaling pathways. Since KRAS inhibition does not affect migration in HCC [9], these data point to potential additional functions of PDE6D (e.g., on migration) beyond its effects on KRAS trafficking in HCC, which could be mediated by the potential novel nuclear PDE6D interactors/effectors identified herein. Accordingly, e.g., E2F1 and HNF4A are known to promote migration in HCC and liver progenitor cells [50,51]. Together, PDE6D might affect diverse cytoplasmatic and nuclear pathways in HCC and potentially also in other cancer types, which should be explored together with its strong potential as a novel therapeutic and diagnostic target in HCC progression and chemoresistance.

## 4. Materials and Methods

### 4.1. Cells and Cell Culture

Primary human hepatocytes (PHH) were isolated and cultured as described in [52]. The human HCC cell lines PLC (ATCC CRL-8024), Hep3B (ATCC HB-8064), Huh-7 (ATCC PTA-4583), and HepG2 (ATCC HB-8065) were used as described in [40]. Sorafenib-resistant (SR) Hep3B (“Hep3B-SR”) and HepG2 (“HepG2-SR”) cells were created by long-term (3–4 months) exposure to sorafenib with stepwise dose escalation (0.5 µM per week) up to 10 µM. In parallel, non-resistant, untreated cells were cultured. As soon as the resistant cells were able to tolerate 8 µM of sorafenib without signs of toxicity, functional assays were performed. Sorafenib (“Nexavar”) was purchased from Selleckchem (Munich, Germany). For some conditions, HCC cells were treated with recombinant human TGF-β1 (purchased from R&D Systems, Minneapolis, MN, USA). The TGF-β-receptor-1 (TGFBR1) inhibitor LY2157299 (“galunisertib”) was obtained from Selleckchem. For pharmacological inhibition of PDE6D signaling, deltarasin was obtained from Cayman Chemicals (Ann Arbor, MI, USA).

### 4.2. Human Material

Paired human HCC tissues and the corresponding non-tumorous liver tissue samples originated from patients that underwent partial hepatectomy. Further, a tissue microarray (TMA) of paraffin-embedded human HCC tissue samples (*N* = 150) was analyzed as described [9,40,41]. Human tissues were obtained and all experimental procedures were performed according to the guidelines of the non-profit state-controlled HTCR (Human Tissue and Cell Research) foundation [41] with informed patients’ consent. Sampling and handling of any patient material was performed in accordance with the ethical principles of the Declaration of Helsinki (HTCR 2015-07).

### 4.3. Immunohistochemistry

Immunohistological analysis was performed as described [9]. Briefly, after deparaffinizing/dewaxing in xylene and rehydration in a graded series of isopropanol, antigen retrieval was achieved by microwave in Tris-EDTA buffer. After, peroxidase block (Dako, Hamburg, Germany) sections were incubated with a validated PDE6D antibody from Thermo Fisher Scientific (Rockford, IL, USA). Next, the slides were washed three times with PBS and then incubated with HRP labelled polymer (conjugated with anti-rabbit secondary antibody), before again washing three times with PBS. Staining was performed with DAB (Dako) followed by counterstaining with hematoxylin (Merck, Darmstadt Germany). KRAS staining was analyzed qualitatively describing “no” (“0”), “low” (“1”), “medium” (“2”), or “strong” (“3”) according to staining intensity and percentage of positive cells (<10%, 10–20%, 20–40%, and >40% positive cells). For correlation analysis and to increase readability, KRAS staining was depicted as “low” (score 0–1) or “high” (score 2–3). KRAS membrane localization was described qualitatively using “no” (“negative”: cytoplasmic/endomembranous staining) or “yes” (“positive”: cells show positive plasma membrane staining). pERK staining was analyzed qualitatively describing “0”: <5%, “1”: 5–20%, “2”: 20–40%, and “3”: more than 20% positive cells. Regarding KRAS, for correlation analysis and to increase readability, pERK staining was depicted as “low” (score 0–1) or “high” (score 2–3). PDE6D staining was analyzed qualitatively describing “low”, “medium”, or “high” (“strong”) staining according to staining intensity and percentage of positive cells (<20%, 20–40%, and >40% positive cells). PDE6D cytoplasmatic localization was described qualitatively using “no” (nuclear staining only or no staining) or “yes” (cytoplasmatic only or cytoplasmatic and nuclear staining).

### 4.4. Immunofluorescence

Immunofluorescence assays were performed as described in [9,53]. In brief, cells were seeded in chamber slides, washed with PBS, fixed with ice cold acetone, and permeabilized using 0.1% TritonX-100 (Sigma-Aldrich, St. Louis, MO, USA). After washing and blocking, the cells were incubated with a specific PDE6D antibody (1 in 200 dilution; Thermo Fisher Scientific) overnight. On the next day, the cells were incubated with a secondary antibody (Alexa-Fluor 488 anti-rabbit IgG; Thermo Fisher Scientific). Next, rinsing with PBS and mounting with Vectashield Hard Set Mounting Medium with DAPI H-1500 (Vector Laboratories, Burlingame, CA, USA) was performed.

### 4.5. RNA Isolation and Reverse Transcription

Total RNA was isolated using E.Z.N.A. Micro Elute Total RNA Kit (Omega, Norcross, GA, USA) as described in [9], and cDNAs of total RNA fractions were generated using the SuperScript II Reverse Transcriptase Kit (Invitrogen, Groningen, Netherlands).

### 4.6. Analysis of mRNA-Expression by Quantitative RT-PCR

Quantitative RT–PCR analysis was performed using a Lightcycler (Roche, Mannheim, Germany) as described in [9]. The following primer pairs were used in this study: 18S rRNA (5′-GCA ATT ATT CCC CAT GAA CG-3′ and 5′-GGG ACT TAA TCA ACG CAA GC-3′), CyclinD1 (5′-GCC TGT GAT GCT GGG CAC TTC ATC TG-3′ and 5′-TTT GGT TCG GCA GCT TGC TAG GTG AC-3′), KRAS (5′-TGG AGC TGG TGG CGT AGG CA-3′ and 5′-AGC CCT CCC CAG TCC TCA TGT-3′), PDE6D (5′-AGG CTA GGG GGA AGG AGA AG-3′ and 5′-CTC GTC CTT GGC TGA CAT GA-3′), S100A4 (5′-GGG CAA AGA GGG TGA CAA GT-3′ and 5′-GCT GCT TAT CTG GGA AGC CT-3′), SNAIL (5′-AGG CCC TGG CTG CTA CA AG-3′ and 5′-ACA TCT GAG TGG GTC TGG AG-3′), and VIMENTIN (5′-AGG AAA TGG CTC GTC ACC TTC GTG AA TA-3′ and 5′-GGA GTG TCG GTT GTT AAG AAC TAG AG CT-3′).

### 4.7. Transfecting Cells with si-RNA-Pools and Plasmid DNA

The Lipofectamine RNAiMax transfection reagent was used (Life Technologies, Darmstadt, Germany) as described in [9]. For siRNA-induced knockdown of PDE6D, a “*si-RNA-pool*-PDE6D” (functionally verified by siTOOLs Biotech GmbH, Planegg, Germany) was used [54]. Overexpression of PDE6D protein in HCC cells was induced by transfection of a human PDE6D open reading frame (ORF) Myc-DDK-tagged plasmid vector (pCMV6-Entry cDNA vector system) from OriGene (Rockville, MD, USA) (CAT#: RC203172) (an according empty control vector without the PDE6D ORF was used as control treatment). Total RNA and protein were isolated 48–72 h after transfection.

### 4.8. Western Blotting

Western blot analysis was performed as described in [9]. The following antibodies were used: anti-GAPDH (1 in 1000 dilution, Santa Cruz Biotechnology, Dallas, TX, USA), anti-histone (1 in 2000 dilution, Cell Signaling Technology, Danvers, MA, USA), anti-KRAS (1 in 1000 dilution; Abcam, Cambridge, UK), anti-beta-actin (1 in 5000 dilution; Sigma-Aldrich), anti-PDE6D (1 in 1000 dilution; Thermo Fisher Scientific), anti-SNAIL (1 in 1000 dilution, Cell Signaling Technology), anti-phospho-ERK (1 in 4000 dilution; Cell Signaling, Frankfurt am Main, Germany) and anti-ERK (1 in 1000 dilution; Cell Signaling). A secondary anti-mouse (1 in 3000 dilution in TBS-T) or anti-rabbit (1 in 3000 dilution in TBS-T) IgG antibody (Chemicon, Hofheim, Germany) was used for detection. Immunoreactions were visualized using NBT/BCIP (Sigma-Aldrich) staining. The protein levels were quantified relative to β-actin expression by performing computer-based densitometry of the scanned western blot images (using the program “ImageJ” (National Institutes of Health, Bethesda, MD, USA)).

### 4.9. Nuclear and Cytoplasmatic Fractioning of HCC Cell Lysates

The subcellular fractionation kit (Abcam) was used as described (http://www.abcam.com/protocols/subcellular-fractionation-protocol).

### 4.10. Functional Analysis of Proliferation, Clonogenicity, and Migration

The xCELLigence System (Roche, Mannheim, Germany) for analysis of cell proliferation (“E-Plates”) was used as described in [55]. Furthermore, cell numbers were counted after seeding of 200,000 cells in 6-wells and 72 hours of transfection with *si-RNA-pools*. Clonogenic assays were performed to analyze the stem cell behavior and attachment dependent colony formation and growth of HCC cells. The assay is based on the ability of a single cell to grow into a colony and was described previously [56]. Cell migration was analyzed using “Boyden chamber assays” as described previously [12].

### 4.11. In silico Analysis

The Oncomine^TM^ cancer microarray database (https://www.oncomine.org/) was used for analysis of PDE6D gene expressions in different datasets as described in the “Results” section of this manuscript. Immunostainings of PDE6D in human tissues were explored using the Human Protein Atlas (https://www.proteinatlas.org/ ENSG00000156973-PDE6D/pathology#top) database. In silico-based analysis of importin-α-dependent nuclear localization signals was performed using the “cNLS Mapper” [42,43]. The cNLS Mapper extracts putative NLS sequences with a score equal to or more than the selected cut-off score value. The score is defined as follows: Higher scores indicate stronger NLS activity. A GUS-GFP reporter protein fused to an NLS with a score of 8, 9, or 10 is exclusively localized to the nucleus. A score of 7 or 8 represents partial nuclear localization, a score of 3, 4, or 5 represents localization to both the nucleus and the cytoplasm, and a score of 1 or 2 represents localization solely to the cytoplasm [42,43]. Analysis of protein-protein interaction datasets was performed using the “Harmonizome” database [44]. Here, protein-protein interactions were derived from low-throughput or high-throughput studies from the following databases: Reactome, NCI Pathways, PhosphoSite, HumanCyc, HPRD, PANTHER, DIP, BioGRID, IntAct, BIND, Transfac, MiRTarBase, Drugbank, Recon X, Comparative Toxicogenomics Database, and KEGG [44]. Gene enrichment-based Kyoto Encyclopedia of Genes and Genomes (KEGG) pathway analysis of potential PDE6D-interacting proteins was performed using the “Harmonizome” database, the “String” database (https://string-db.org/) and the “DAVID Bioinformatics” database. The “Enrichr” database [49] was used to describe enriched transcription factor terms for PDE6D-interacting proteins using different datasets (“Enrichr Submissions TF-Gene Coocurrence” and “Transcription Factor PPIs”).

### 4.12. Statistical Analysis

Results are expressed as mean ± SEM. The Student’s *t*-test or one-way ANOVA, if appropriate, were used for comparisons between groups. The level of significance was *p* < 0.05 (using the abbreviations “ns”, not significant, and “*”, *p* < 0.05). The number of independent experiments was *n* ≥ 3 (if not depicted otherwise). Calculations were performed using GraphPad Prism software (GraphPad Software, Inc., San Diego, CA, USA) and SPSS (SPSS Statistics 23, IBM Corp., Armonk, NY, USA).

## 5. Conclusions

In this study, we newly showed that the *delta subunit of rod-specific photoreceptor cGMP phosphodiesterase* (PDE6D) contributes to hepatocellular carcinoma progression. We found that PDE6D mRNA and protein expression levels were overexpressed in HCC. Furthermore, our results showed a clear correlation of PDE6D expression and cellular localization with tumor grading, tumor stages, ERK activation, and KRAS activation. Moreover, TGF-β1-TGF-β receptor signaling was identified to drive PDE6D upregulation. Functionally, PDE6D induced migration, proliferation, and sorafenib resistance in HCC. Together, our results demonstrated that targeting PDE6D outlines a potential novel therapeutic strategy for HCC in the future.

## Supplementary Materials

The following are available online at https://www.mdpi.com/2072-6694/11/3/398/s1, Figure S1: PDE6D-mediated ERK-activation in HCC; Figure S2: Snail protein expression after TGF-β1-mediated stimulation of HCC cells; Figure S3: KRAS expression and membrane localization in human HCC tissues as correlated with PDE6D expression and cytoplasmatic localization; Figure S4: Potential protein-interactions of PDE6D and PDE6D-interactome dependent pathways; Figure S5: Potential transcription factor interactions of the PDE6D-interactome.
