# Supplementary material for: The Delta Subunit of Rod-Specific Photoreceptor cGMP Phosphodiesterase (PDE6D) Contributes to Hepatocellular Carcinoma Progression
Source: Cancers (Basel). 2019 Mar 21;11(3):398. doi: 10.3390/cancers11030398 (PMC6468542; doi:10.3390/cancers11030398)
Supplement: Supplementary file 1 [file cancers-11-00398-s001.pdf]

cancers

Article

The delta subunit of rod-specific photoreceptor cGMP phosphodiesterase (PDE6D) contributes to hepatocellular carcinoma progression

Peter Dietrich, Claus Hellerbrand and Anja Bosserhoff

Supplementary Material

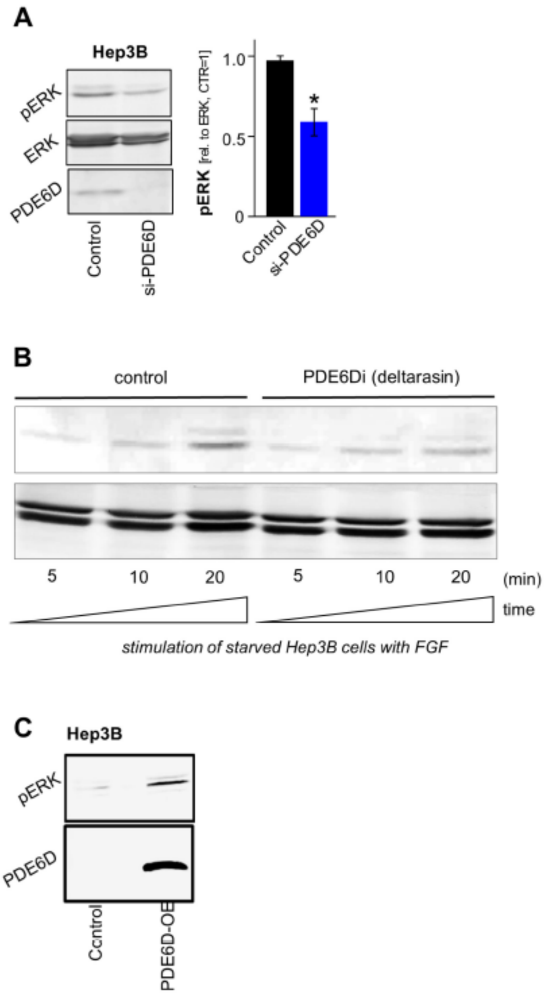

Figure 1. PDE6D-mediated ERK-activation in HCC. (**A**) Exemplary image (right side) and densitometric analysis (left side) of ERK-activation (pERK/ERK-levels) (Western blot analysis) in Hep3B cells after si-RNA-mediated PDE6D-suppression (*: $p < 0.05$ vs control). (**B**) Western blot analysis (exemplary image representing two independent experiments) revealing time-dependent recombinant Fibroblast-growth-factor (FGF)-induced ERK-activation (pERK/ERK-levels) in starved HCC cells (Hep3B) (cells were starved for 24 hours prior to stimulation with FGF) was prevented by co-treatment with the pharmacologic inhibitor of the PDE6D-KRAS-interaction deltarasin (5 µM). (**C**) Phospho-ERK (pERK) and PDE6D levels after forced PDE6D overexpression in sorafenib-resistant HCC cells (Hep3B).

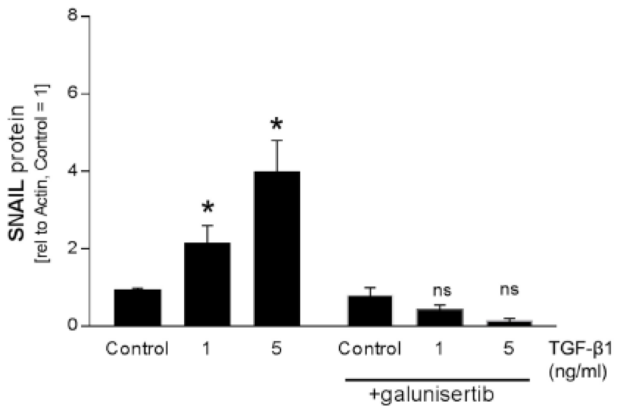

Figure S2. Snail protein expression after TGF-β1-mediated stimulation of HCC cells. Densitometric analysis of Snail protein expression levels (Western blot analysis) in PLC cells that were stimulated with different doses (0, 1, 5 ng/ml) of recombinant human TGF-β1 for 72 hours, with or without co-treatment with 15 μM of the TGF-β-receptor-1 (TGFBR1) inhibitor LY2157299 ("galunisertib") (the densitometric values represent two independent Western blot analysis) (*: $p < 0.05$ vs control; ns: non-significant vs control).

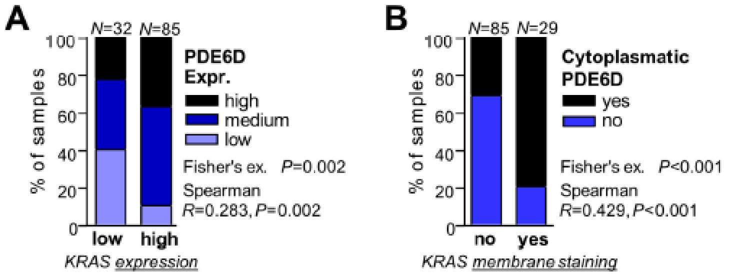

Figure S3. KRAS expression and membrane localization in human HCC tissues as correlated with PDE6D expression and cytoplasmatic localization. (**A**) Tissue micro array analysis of PDE6D expression levels (high, medium, low) in human HCC tissues correlated with KRAS expression (high, low). (**B**) Tissue micro array analysis comparing KRAS membrane staining ("yes" vs "no") in human HCC tissues with ("yes") and without ("no") cytoplasmatic localization pattern of PDE6D.

A

symbol	localization: nuclear	localization: non-nuclear	HCC-related (PMIDs)	Pathway/Function
ARL15				Signaling, Ras-family
ARL2			27798868	Signaling, Ras-family
ARL3				Signaling, Ras-family
CDC42			25978354	Cell cycle, mitosis
CETN3				Cell cycle, mitosis
COPS5			27524414	Signaling
CUL1			26097587	Protein degradation
→ **E2F1**			28474358; 28134624	**TF**
FAM219A				nd
GNAI1			23691483	Signaling
GRK1				Rhodopsin-signaling
GRK7				Rhodopsin-signaling
→ **HNF4A**			29566023; 28498607	**TF**
**HRAS**			29423069	Signaling
**KRAS**			29275358	Signaling
→ **MAX**			29740493	**TF**
→ **MYC**			29893492	**TF**
PDE6A				Rhodopsin-signaling
PDE6B				Rhodopsin-signaling
PTGIR				nd
RAB13				Signaling, Ras-family
RAB18			23471881	Signaling, Ras-family
RAB8A				Signaling, Ras-family
RAD23A				Signaling, Ras-family
RAP1A			27780730	Signaling, Ras-family
RAP2B			28081729	Signaling, Ras-family
RASA1			26126858	Signaling, Ras-family
RHEB			29467900	Signaling, Ras-family
RHOA			29954442	Signaling, Ras-family
RHOB			28042950	Signaling, Ras-family
RND1				Signaling, Ras-family
RPGR				Signaling, Ras-family
→ **TAF1**				**TF-associated**
UBC				Protein degradation

B

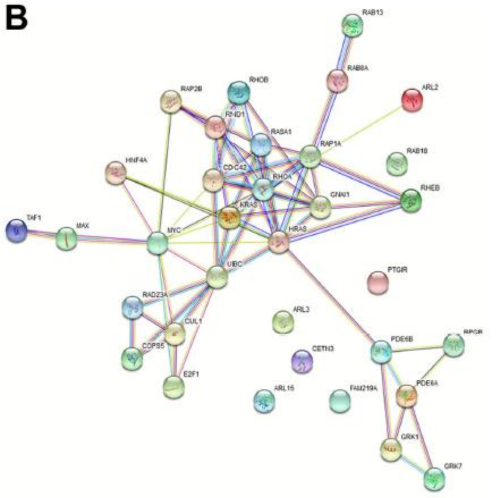

KEGG Pathways			
pathway ID	*pathway description*	*count in gene set*	*false discovery rate*
04744	Phototransduction	4	2.25e-05
04062	Chemokine signaling pathway	6	6.29e-05
04360	Axon guidance	5	0.000168
04010	MAPK signaling pathway	6	0.000233
04144	Endocytosis	5	0.000738
05200	Pathways in cancer	6	0.000738
04014	Ras signaling pathway	5	0.00112
05219	Bladder cancer	3	0.00112
04722	Neurotrophin signaling pathway	4	0.00123
05206	MicroRNAs in cancer	4	0.00259
05211	Renal cell carcinoma	3	0.00425
05220	Chronic myeloid leukemia	3	0.00488
04350	TGF-beta signaling pathway	3	0.00623
04015	Rap1 signaling pathway	4	0.00654
04510	Focal adhesion	4	0.00654
05222	Small cell lung cancer	3	0.00654
04972	Pancreatic secretion	3	0.00722
05205	Proteoglycans in cancer	4	0.00722
04660	T cell receptor signaling pathway	3	0.00865

Figure 4. Potential protein-interactions of PDE6D and PDE6D-interactome dependent pathways. (**A**) Analysis of protein-protein interaction datasets was performed using the "Harmonizome" database. The table depicts potential PDE6D-interacting proteins that were derived from low-throughput or high-throughput studies from the following databases: Reactome, NCI Pathways, PhosphoSite, HumanCyc, HPRD, PANTHER, DIP, BioGRID, IntAct, BIND, Transfac, MiRTarBase, Drugbank, Recon X, Comparative Toxicogenomics Database, and KEGG. The table also depicts information about cellular localization, HCC-relation according to literature (Pubmed-IDs) and related pathways/functions. The black arrows mark nuclear proteins. (**B**) String database and DAVID bioinformatics database-derived graphical illustration (top panel) as well as Gene enrichment based Kyoto Encyclopedia of Genes and Genomes (KEGG) pathway analysis (bottom panel) of the PDE6D-interactome.

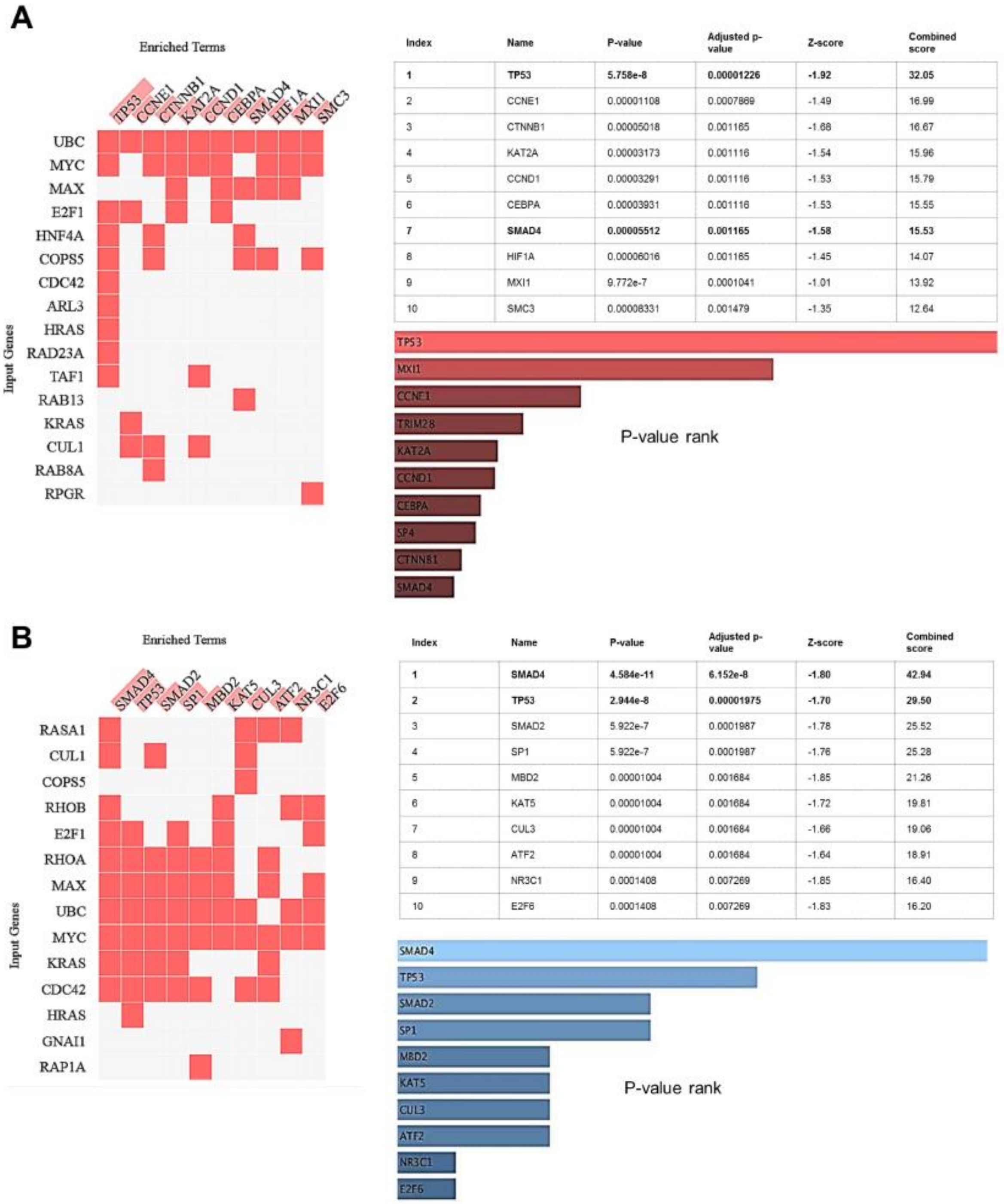

A

Index	Name	P-value	Adjusted p-value	Z-score	Combined score
**1**	**TP53**	**5.758e-8**	**0.00001226**	**-1.92**	**32.05**
2	CCNE1	0.00001108	0.0007869	-1.49	16.99
3	CTNNB1	0.00005018	0.001165	-1.68	16.67
4	KAT2A	0.00003173	0.001116	-1.54	15.96
5	CCND1	0.00003291	0.001116	-1.53	15.79
6	CEBPA	0.00003931	0.001116	-1.53	15.55
**7**	**SMAD4**	**0.00005512**	**0.001165**	**-1.58**	**15.53**
8	HIF1A	0.00006016	0.001165	-1.45	14.07
9	MXI1	9.772e-7	0.0001041	-1.01	13.92
10	SMC3	0.00008331	0.001479	-1.35	12.64

B

Index	Name	P-value	Adjusted p-value	Z-score	Combined score
**1**	**SMAD4**	**4.584e-11**	**6.152e-8**	**-1.80**	**42.94**
**2**	**TP53**	**2.944e-8**	**0.00001975**	**-1.70**	**29.50**
3	SMAD2	5.922e-7	0.0001987	-1.78	25.52
4	SP1	5.922e-7	0.0001987	-1.76	25.28
5	MBD2	0.00001004	0.001684	-1.85	21.26
6	KAT5	0.00001004	0.001684	-1.72	19.81
7	CUL3	0.00001004	0.001684	-1.66	19.06
8	ATF2	0.00001004	0.001684	-1.64	18.91
9	NR3C1	0.0001408	0.007269	-1.85	16.40
10	E2F6	0.0001408	0.007269	-1.83	16.20

Figure S5. Potential transcription factor interactions of the PDE6D-interactome. Gene enrichment analysis using the "Enrichr" database depicting significantly enriched transcription factor terms for the 34 PDE6D-interacting protein list (**Figure S1**) in the "Enrichr Submissions TF-Gene Coocurrence" dataset (**A**) and the "Transcription Factor PPIs" dataset (**B**).
